# Enhanced Action Recognition Using Multiple Stream Deep Learning with Optical Flow and Weighted Sum

**DOI:** 10.3390/s20143894

**Published:** 2020-07-13

**Authors:** Hyunwoo Kim, Seokmok Park, Hyeokjin Park, Joonki Paik

**Affiliations:** Department of Image, Graduate School of Advanced Imaging Science, Multimedia and Film, Chung-Ang University, Seoul 06974, Korea; hyunwoo@ipis.cau.ac.kr (H.K.); seokmok@ipis.cau.ac.kr (S.P.); parkjin@ipis.cau.ac.kr (H.P.)

**Keywords:** action recognition, score fusion, multi-stream, deep learning

## Abstract

Various action recognition approaches have recently been proposed with the aid of three-dimensional (3D) convolution and a multiple stream structure. However, existing methods are sensitive to background and optical flow noise, which prevents from learning the main object in a video frame. Furthermore, they cannot reflect the accuracy of each stream in the process of combining multiple streams. In this paper, we present a novel action recognition method that improves the existing method using optical flow and a multi-stream structure. The proposed method consists of two parts: (i) optical flow enhancement process using image segmentation and (ii) score fusion process by applying weighted sum of the accuracy. The enhancement process can help the network to efficiently analyze the flow information of the main object in the optical flow frame, thereby improving accuracy. A different accuracy of each stream can be reflected to the fused score while using the proposed score fusion method. We achieved an accuracy of 98.2% on UCF-101 and 82.4% on HMDB-51. The proposed method outperformed many state-of-the-art methods without changing the network structure and it is expected to be easily applied to other networks.

## 1. Introduction

Action recognition is one of the representative tasks in the video understanding field. It aims to recognize human actions from the frames of video, and it is mainly applied to intelligent surveillance systems using closed-circuit television (CCTV) to detect abnormal behaviors, such as assault and theft. Because action recognition uses video instead of a single image as input, it generally requires a huge amount of computation. For the same reason, analysis of spatial information is not easy because the view point can change over time. To solve these problems, it is necessary to analyze temporal information across multiple frames in order to understand the relationship between adjacent frames.

Various conventional action recognition algorithms have been proposed in the literature using: (i) spatial analysis by convolution for each frame and temporal analysis using a long short-term memory (LSTM) [1], (ii) three-dimensional (3D) convolution neural network with a spatio-temporal kernel extended to the time axis [2], and (iii) two-stream method using RGB frames and optical flow frames [3,4]. In the field of action recognition, most state-of-the-art methods use both 3D convolution and multi-stream structure [5,6,7,8,9]. In addition, they use a pre-trained network on large video dataset, such as Kinetics [10]. Recently, various methods using human pose information with three or more multi-streams were proposed [6,9]. These conventional methods using optical flow estimation make the network difficult to learn flows of the main objects in input frames, because the flow images contain a lot of noise and flows of background. Alternatively, various attention-based methods have been proposed to make the action recognition network focus the on main region while training [11,12].

In this paper, we present a method that helps to efficiently train the network by emphasizing the movements of the main objects by applying image segmentation using DeepLabV3 [13]. In addition, we present a combination method using a weighted sum that can be applied instead of concatenation or summation in the conventional multi-stream network structure. Because the proposed method is not based on human-based information, its application can extend in many areas, such as video action recognition. The proposed method can be easily applied to many other existing networks to increase the accuracy without major modification of the baseline network.

## 2. Related Works

### 2.1. Action Recognition

Although traditional action recognition methods used various image processing techniques, such as histogram of optical flow or oriented gradients, deep neural networks recently replaced the role of extracting features, and end-to-end recognition [14,15,16]. In the field of deep learning-based action recognition, various studies have been conducted to effectively analyze both spatial and temporal information. Karpathy et al. analyzed temporal information of video using long short-term memory (LSTM) after analyzing space information by convolution for each frame [1]. Tran et al. proposed a deep three-dimensional convolutional networks (3D ConvNets) that can help the convolution kernel to learn not only spatial information, but also temporal information at the same time using the spatio-temporal feature kernels [2]. Simonyan et al. proposed a two-stream network using both RGB and optical flow as input [4]. Feichtenhofer et al. proposed a three-dimensional fused two-stream network that applies 3D ConvNets to two-stream two-dimensional (2D) ConvNets results [17].

Optical flow estimation was used to visualize the motion of objects in a video based on information of adjacent frames. Frames created using optical flow estimation can greatly help the network to analyze temporal information, thereby improving the accuracy. Therefore, most of the latest action recognition techniques are based on both multi-stream structures and 3D ConvNets.

Weakly-supervised learning-based methods have also been proposed for action recognition and localization. Nguyen et al. proposed a method using attention-model based on weakly-supervised learning [18]. Nguyen’s method uses an attention model to distinguish between foreground and background. Liu et al. proposed a contrast-based localization evaluation network with an action proposal evaluator to generate pseudo-labels [19]. Zhang et al. proposed a knowledge transfer framework using an encoder-decoder network [20].

### 2.2. Trend of Advanced Deep Learning-Based Action Recognition Techniques

**Figure 1:**
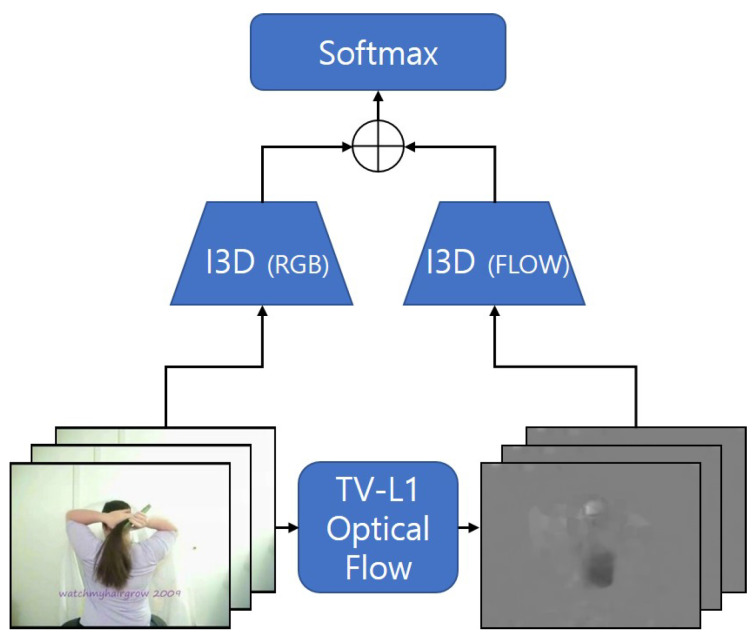
The overall architecture of I3D.

Joao Carreira et al. proposed Inflated 3D ConvNet (I3D) and released huge datasets, called Kinetics [5,10]. The overall architecture of conventional I3D is shown in Figure 1. In addition, the authors proposed a method to pre-train 3D ConvNets with a 2D image dataset, such as ImageNet by inflating the convolution kernel after training a 2D network [21]. I3D applies a two-stream structure for RGB and optical flow to the Inception-v1 along with the 3D convolution [22]. Similar to other multi-stream methods, it adds the results of each stream to form a final score, and finally performs action recognition based on the final score. Although I3D outperforms most conventional methods, it is not reasonable to combine them one-on-one, ignoring the different accuracy of the RGB and optical flow streams.

Vasileios Choutas et al. proposed a Pose MoTion Representation (PoTion) using I3D as the base network [6]. PoTion first estimates the poses in each frame through the pose estimation model, and then visualizes the flows of the main parts of the person, such as hand, head, and foot. The visualized flow was added to the input of I3d to construct three-stream for improved performance. Because this method requires additional calculation of human pose, its application is limited to human action recognition.

Wang et al. also proposed an I3D-based action recognition method, where the four-stream, including the 1st order Fisher vector, 2nd order Fisher vector, bag-of-words, and high abstraction feature, takes the results of the two-stream I3D as input, and performs the normalization process [7]. The normalized feature vectors are concatenated and enters the prediction network as input to perform action recognition. Because four streams and one prediction networks were added, Wang’s method showed the state-of-the-art performance on HMDB-51 datasets at the cost of increased amount of computation [23].

Crasto et al. proposed motion-augmented RGB Stream (MARS) that performed self-training using a teacher–student method to mimic an optical flow image by itself using only RGB images [8]. Crasto’s method requires optical flow images to train the imitated model to provide high-accuracy inference without optical flow computation.

Stroud et al. also proposed distilled 3D network(D3D) using the teacher-student method without optical flow during the inference process [24].

Yan et al. proposed Pose-Action 3D Machine (PA3D) that first creates different pose modalities of pose heat maps (joints, part affinity fields, convolutional features) through spatial pose CNN from the input [9]. For each generated pose heatmap, the spatio-temporal pose heatmap is calculated through temporal pose convolution. Finally, Action CNN takes the result as input, generates three scores, and then fuses the scores.

### 2.3. Attention-Based Action Recognition Techniques

Attention mechanism makes the network focus on the main regions of the input data. The attention mechanism with Encoder-Decoder structure was originally proposed for machine translation [25,26]. Later, Xu et al. divided a visual attention into hard attention and soft attention for image captioning [27]. Unlike hard attention, soft attention is a deterministic mechanism that uses all of the features in the image for end-to-end learning [28].

In the field of action recognition, various methods have been proposed to apply soft attention so that the network can focus on main regions. Sharma et al. proposed a method that performs action recognition based on the soft attention by applying a structure using Recurrent Neural Network (RNN) and LSTM [11]. Sharma’s method could enhance the accuracy, but it is difficult to apply to other networks, and the use of LSTM decreases the accuracy. Girdhar et al. proposed a method for the network to focus on specific parts while training [12]. Girdhar’s method combined a bottom-up saliency with a top-down attention to provide factorization of the attentional processing. Li et al. proposed an attention mechanism-based deformable module to efficiently analyze long-term time information [29].

Unlike the conventional attention-based methods, we present a method to help the network focus on the main region by using image segmentation rather than attention module. The proposed method is applied to optical flow frames rather than RGB frames to enhance not only spatial information of the main object, but also temporal information.

### 2.4. Image Segmentation

Various image segmentation approaches are classified into semantic and instance segmentations. The former classifies all of the pixels of an image into a specific class without object classification, whereas the latter performs object classification. DeepLabV3 is one of the representative methods proposed in the field of semantic segmentation [13]. It consists of three types: encoder using atrous convolution to Resnet, decoder using atrous spatial pyramid pooling, and bilinear upsampling.

In this paper, we present a method that can enhance the flow information of the main objects in the optical flow frame based on semantic segmentation process while using the DeepLabV3 model.

## 3. Proposed Methods

**Figure 2:**
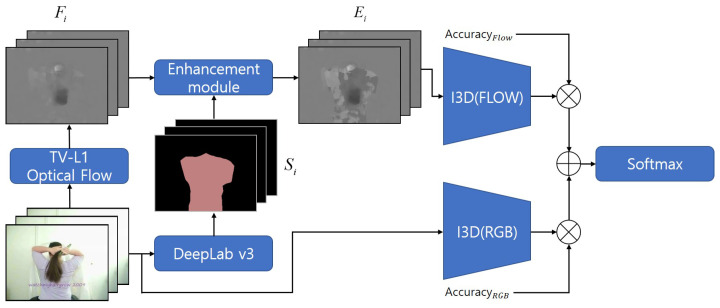
The overall architecture of proposed method.

In this section, we present a method to emphasize the flow of the main objects in optical flow frames using Image Segmentation to improve the performance of action recognition. Additionally, we present a simple but effective score fusion method that can be applied to conventional methods that are based on multi-stream structure. Figure 2 shows the overall architecture of the proposed method.

### 3.1. Optical Flow Enhancement

**Figure 3:**
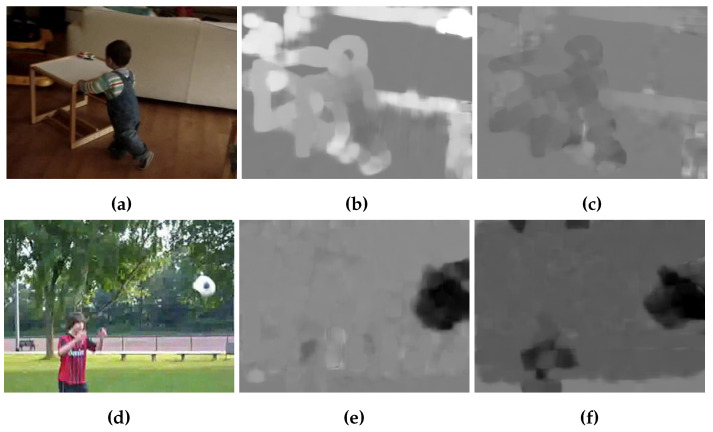
Images of TV-L1 optical flow. (**a**,**b**) shows original RGB images. (**d**,**e**) shows x-axis TV-L1 optical flow images. (**c**,**f**) shows y-axis images

Recently, most of deep learning based action recognition methods use optical flow, such as TV-L1, as an additional input so that the network can effectively analyze temporal information of video [3]. Optical flow is effective for analyzing the temporal information of video, but it has a disadvantage in that the background flow, including noise, makes it difficult to identify main objects in an optical flow image as shown in Figure 3. Figure 3a,d show two original RGB images from the HMDB-51 dataset. Figure 3b,e show TV-L1 optical flow images representing x-axis motions, and Figure 3c,f show y-axis motions. In this subsection, we propose a novel method that can emphasize the flow of main objects to overcome the shortcomings of optical flow.

The proposed method first segments the main objects from the original RGB frame while using DeepLabV3 [13]. Next, the segmented frame is compared with the optical flow frame, and the pixels in the main object region are then located in the optical flow frame that is enhanced by the α value. Pixels where the main objects are not located or where there is no flow are maintained without enhancement. The proposed method is expressed as

(1) $$E_{i,x,y}=\left\{\begin{array}{cc} F_{i,x,y}, & \mathrm{if}\;S_{i,x,y}=0\;\mathrm{or}\;F_{i,x,y}=128\\ F_{i,x,y}\cdot \left(1+\alpha \right), & \mathrm{else}\;\mathrm{if}\;F_{i,x,y}>128\\ F_{i,x,y}\cdot \left(1-\alpha \right), & \mathrm{otherwise}\; \end{array}\right.$$

where $S_{i}$ represents the segmented image of frame *i*, $F_{i}$ represents the optical flow image of frame *i*, and $E_{i}$ represents the enhanced flow image of frame *i*.

**Figure 4:**
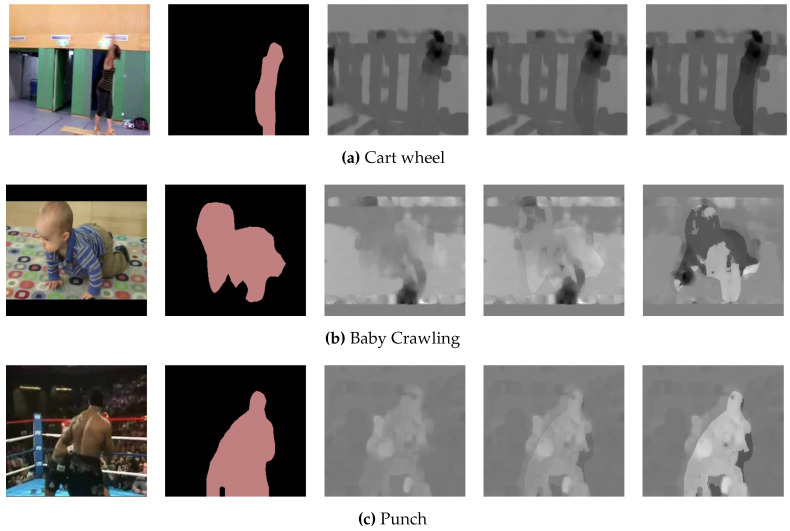

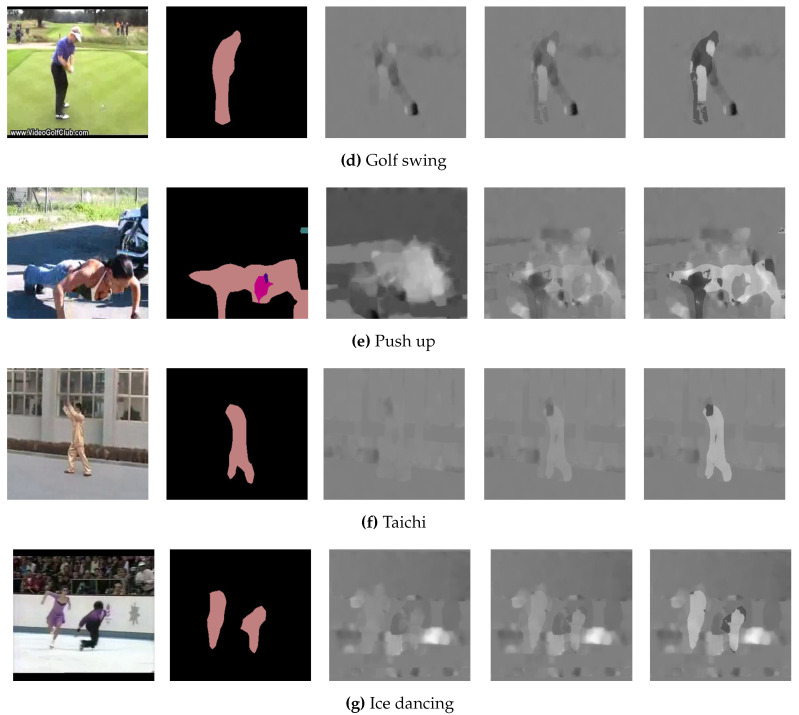
Enhanced images by proposed method.

Figure 4 shows enhanced optical flow images while using the proposed method. The first and second columns show the original RGB images and the segmented images by DeepLabV3, respectively. The third column shows the original TV-L1 optical flow images, and columns 4 and 5 show enhanced optical flow images using the proposed method with alpha values set to 0.1 and 0.3, respectively. We can see that the pixels of the main objects are enhanced proportional to alpha value.

### 3.2. Score Fusion Method

Most of deep learning based action recognition methods have applied a multi-stream structure to effectively analyze spatio-temporal information. In the multi-stream structure, after training each stream, a score fusion process is performed in order to calculate a single score. In most of conventional methods, score fusion was performed by summation or averaging individual stream [4]. This conventional method has a problem of fused one-on-one while ignoring the different accuracy of each stream.

**Figure 5:**
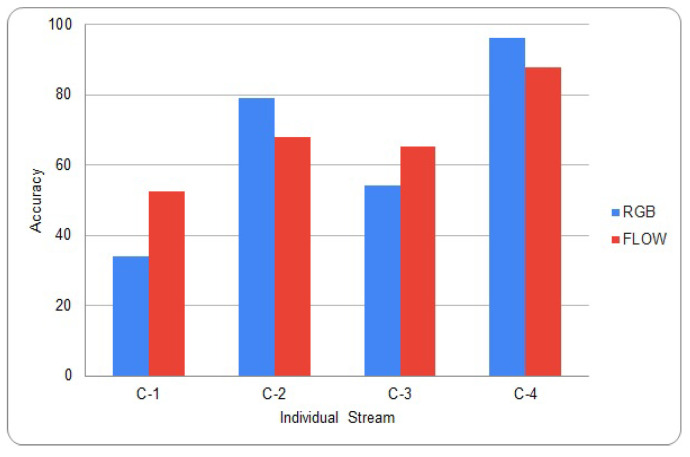
Inference result on UCF-101 and HMDB-51.

Many different methods have been proposed to give an appropriate weight to each stream when multiple-streams are fused together [30]. Nandakumar et al. proposed a method using the likelihood ratio [31]. Srivastava et al. proposed a method using deep Boltzmann machines [32]. Neverova et al. proposed a method using the modality dropping process [33]. Because these conventional methods are based on training, there are many calculations required. The proposed score fusion method simply calculates relative weights. We can perform inference with a single trained stream before score fusion process. The graph in Figure 5 shows the inference results on the HMDB-51 and UCF-101 validation set for each individual stream [23,34]. C-1 and C-2 represent the results of HMDB-51, and C-3 and C-4 represent the results of UCF-101. C-2 and C-4 were pre-trained on both ImageNet and kinetics, and the rest trained from scratch [10,21]. From the graph, we can see the deviation of accuracy between each stream is large, which should be sufficiently considered in the score fusion process.

Therefore, the proposed method performs score fusion with different rates by referring to the accuracy of each stream. The proposed method does not require an additional training process and it can be easily applied to all conventional multi-stream based network.

The proposed method first trains network streams in the same way as conventional methods, such as I3D [5]. Next, accuracy is calculated by performing inference on validation sets for each trained stream. When performing inference in a multi-stream method, score fusion is performed by weighted sum of accuracy as the weight for each stream result. Equation (2) shows the case where the proposed score fusion method is applied to the conventional two-stream network.

(2) $$Score_{RGB+Flow}=\left(Accuracy_{RGB}\cdot Score_{RGB}\right)+\left(Accuracy_{Flow}\cdot Score_{Flow}\right)$$

## 4. Results

### 4.1. Datasets and Metrics

To evaluate the performance of the proposed methods, training and testing were performed on action recognition datasets including UCF-101 [34] and HMDB-51 [23]. UCF-101 consists of 101 action classes with 13,320 videos collected from YouTube. HMDB-51 consists of 51 action classes with 7000 videos, mostly from movies. UCF-101 and HMDB-51 both provide three test/train splits. All experimental results are averaged over three splits. To apply the proposed methods for experiments, we need to construct a validation set. To this end, we randomly constructed 15% of the train set as a validation set. To perform image segmentation in the proposed method, we used the DeepLabV3 model trained with PASCAL VOC 2012 [35]. PASCAL VOC 2012 consists of 20 object classes, including human.

For ImageNet and Kinetics, we used the pre-trained model provided by I3D [5,10,21]. Therefore, all models in this section are pre-trained by the conventional I3D with α = 0.

### 4.2. Comparison on Various Alpha Values

We evaluated the method proposed in Section 3.1 by applying it to Two-Stream I3D [5]. We used the value of Alpha of 0, 0.1, 0.15, and 0.3 and recorded the corresponding accuracy measure. Table 1 and Table 2 show top-1 and top-3 accuracies, depending on the alpha value, dataset, and the pre-training status. The pre-trained network in both ImageNet and kinetics dataset and whose alpha value was set to 0.1 showed the highest accuracy with 98.1% in UCF-101 and 82.2% in HMDB-51 [23,34].

In the experiment of the pre-trained network, when the alpha value was set to 0.3 or 0.15, the proposed method gave a lower accuracy than the conventional method. However, all of the networks trained from scratch showed higher accuracy than the conventional method. When the alpha value was set to 0.1, regardless of whether it was pre-trained, it showed the highest accuracy. Experiments show that the flow of the main object is emphasized while maintaining the information of other flows when the alpha value is properly set.

### 4.3. Experiment about Proposed Score Fusion Method

We evaluated the score fusion method proposed in Section 3.2. For experiments, inference was performed on validation set for individual streams (RGB, α = 0, 0.1, 0.15, 0.3) to set the weights by computing accuracy. Table 3 and Table 4 show the experimental results of applying the proposed score fusion method to two-stream I3D, and column 2 shows the weights that are based on their validation accuracy. Averaging was used for the score fusion method of the conventional method in both Table 3 and Table 4. As a result of the experiment, the proposed method can obtain a proper set of weights by reflecting the accuracy of an individual stream, which proves that the proposed method outperforms conventional methods. As a result of the experiment, the proposed method outperformed conventional methods in most cases. In some cases, top-1 accuracy was not improved, but top-3 accuracy was improved. Therefore, it can be proved that the proposed score fusion method more effectively reflects the accuracy of each stream than the conventional method.

### 4.4. Comparison with State-of-the-Art Methods

We evaluated two action recognition data sets, including UCF-101 and HMDB-51, in order to compare the proposed methods with conventional state-of-the-art methods. In the proposed method, the alpha value was set to 0.1 as shown in Table 5. The accuracy of the comparison target was referenced from the corresponding paper.

We have achieved higher accuracy than conventional methods on UCF-101 dataset. Table 6 shows comparison results with conventional methods on the UCF-101 dataset. LGD-3D Two-stream and PoTion + I3D showed similar accuracies to that of the proposed method, but the accuracy of the proposed method was higher on other datasets [6,36].

The proposed method showed higher accuracy than most other conventional methods on HMDB-51 dataset. Table 7 shows the accuracy (averaged over three splits) comparison with conventional methods on HMDB-51. The proposed method was applied to two-stream I3D, recording 82.4% accuracy, showing a similar result to HAF + BoW/FV halluc, a method with more streams [7].

## 5. Discussion and Conclusions

In this paper, we presented an optical flow enhancement method while using image segmentation that can be used in the field of video analysis, including action recognition. The proposed method enhances the flow of the main object in the optical flow frame and helps the network to focus on the main object. Unlike the conventional attention-based method, the proposed method is applied to optical flow frames to make the network analyze both spatial and temporal information. In addition, the enhancement of the main object region can be adjusted by setting the alpha value. Furthermore, we presented a validation accuracy-based score fusion method that can be applied to many conventional multi-stream-based networks. The proposed method can easily compute the weight and gives a higher accuracy than conventional methods. As a result of using the proposed method on conventional I3D, the proposed method outperformed most conventional action recognition methods in the sense of an accuracy measure. Because the proposed method does not need to change the network structure, its application is not limited to I3D.

## Figures and Tables

**Table 1:** Experimental result of changing alpha value on UCF-101.

Alpha	Pre-Train	Acc-Top1	Acc-Top3
0 (I3D)	ImageNet, Kinetics	98.0	99.9
0.1	ImageNet, Kinetics	98.1	99.9
0.15	ImageNet, Kinetics	97.8	99.8
0.3	ImageNet, Kinetics	97.9	99.9
0 (I3D)	-	74.4	89.0
0.1	-	76.1	90.7
0.15	-	75.3	90.2
0.3	-	75.6	90.7

**Table 2:** Experimental result of changing alpha value on HMDB-51.

Alpha	Pre-Train	Acc-Top1	Acc-Top3
0 (I3D)	ImageNet, Kinetics	81.8	93.1
0.1	ImageNet, Kinetics	82.2	93.6
0.15	ImageNet, Kinetics	81.5	93.3
0.3	ImageNet, Kinetics	81.2	93.3
0 (I3D)	-	54.5	76.3
0.1	-	57.3	77.4
0.15	-	56.4	77.4
0.3	-	56.3	77.0

**Table 3:** Experiment about Score Fusion on UCF-101.

Alpha	Weight (RGB/FLOW)	Pre-Train	Acc-Top1	Acc-Top3
0	1/1	ImageNet, Kinetics	98.0	99.9
0	0.96/0.91	ImageNet, Kinetics	98.1	99.9
0.1	0.96/0.93	ImageNet, Kinetics	98.2	99.9
0.15	0.96/0.93	ImageNet, Kinetics	97.8	99.8
0.3	0.96/0.93	ImageNet, Kinetics	97.9	99.9
0	1/1	x	74.4	88.9
0	0.54/0.66	x	74.8	89.7
0.1	0.54/0.66	x	76.5	91.2
0.15	0.54/0.65	x	75.6	90.8
0.3	0.54/0.65	x	76.4	91.2

**Table 4:** Experiment about Score Fusion on HMDB-51.

Alpha	Weight (RGB/FLOW)	Pre-Train	Acc-Top1	Acc-Top3
0	1/1	ImageNet, Kinetics	81.8	93.1
0	0.79/0.68	ImageNet, Kinetics	82.1	93.4
0.1	0.79/0.73	ImageNet, Kinetics	82.4	93.7
0.15	0.79/0.71	ImageNet, Kinetics	81.7	93.9
0.3	0.79/0.68	ImageNet, Kinetics	81.2	93.3
0	1/1	x	54.5	76.3
0	0.34/0.45	x	55.2	76.7
0.1	0.34/0.51	x	57.3	78.5
0.15	0.34/0.51	x	57.1	77.8
0.3	0.34/0.52	x	57.6	77.9

**Table 5:** Ablation Study.

Ablation Study (Averaged Score Fusion/ Proposed Score Fusion Method)				
DATASET	Alpha Value			
	0	0.1	0.15	0.3
UCF-101 pre-trained	98.0/98.1	98.1/98.2	97.8/97.8	97.9/97.9
HMDB-51 pre-trained	81.8/82.1	82.2/82.4	81.5/81.7	81.2/81.2
UCF-101	74.4/74.8	76.1/76.5	75.3/75.6	75.6/76.4
HMDB-51	54.5/55.2	57.3/57.3	56.4/57.1	56.3/57.6

**Table 6:** Comparison with State-of-the-Art Methods on UCF-101 (Accuracy Top-1).

UCF-101	
Metric	Accuracy
Two-Stream [4]	88.0
Two-Stream Fusion + IDT [17]	93.5
STDA-ResNeXt-101 [29]	95.5
AE-I3D [37]	95.9
STM [38]	96.2
CMA iter1-S [39]	96.2
Hidden Two-Stream [40]	97.1
CCS + TSN [41]	97.4
D3D Ensemble [24]	97.6
HATNet [42]	97.8
Two-Stream I3D [5]	98.0
MARS + RGB + Flow [8]	98.1
PoTion + I3D [6]	98.2
LGD-3D Two-Stream [36]	98.2
Ours	98.2

**Table 7:** Comparison with State-of-the-Art Methods on HMDB-51 (Accuracy Top-1).

HMDB-51	
Metric	Accuracy
Two-Stream [4]	59.4
Two-Stream Fusion + IDT [17]	69.2
Sharama’s Method [11]	71.3
STDA-ResNeXt-101 [29]	72.7
AE-I3D [37]	74.7
HATNet [42]	76.5
Hidden Two-Stream [40]	78.7
LGD-3D Two-Stream [36]	80.5
D3D Ensemble [24]	80.5
Two-Stream I3D [5]	80.9
MARS + RGB + Flow [8]	80.9
PoTion + I3D [6]	80.9
CCS + TSN [41]	81.9
EvaNet [43]	82.1
PA3D [9]	82.1
HAF + BoW/FV halluc. [7]	82.48
Ours	82.4
